# Supplementary material for: The problem is escalating: Barriers faced by medical students in conducting research; A scoping literature review
Source: PLoS One. 2026 Feb 27;21(2):e0343232. doi: 10.1371/journal.pone.0343232 (PMC12948103; doi:10.1371/journal.pone.0343232)
Supplement: S2 Appendix — (DOCX) [file pone.0343232.s002.docx]

**S2 Appendix.** Search strategy

| **Database** | **Search terms** | **Number of studies retrieved** |
| --- | --- | --- |
| **PubMed** | ("medical students"[Title/Abstract] OR "undergraduate medical education"[Title/Abstract]) AND ("research barriers"[Title/Abstract] OR "research challenges"[Title/Abstract] OR "research participation"[Title/Abstract] OR "research obstacles"[Title/Abstract] OR "mentorship"[Title/Abstract] OR "funding"[Title/Abstract] OR "time constraints"[Title/Abstract]) | 350 |
| **Scopus** | TITLE-ABS-KEY("medical students" OR "undergraduate medical education") AND TITLE-ABS-KEY("research barriers" OR "research challenges" OR "research participation" OR "research obstacles" OR "mentorship" OR "funding" OR "time constraints") | 489 |
| **Web of Science** | TS=("medical students" OR "undergraduate medical education") AND TS=("research barriers" OR "research challenges" OR "research participation" OR "research obstacles" OR "mentorship" OR "funding" OR "time constraints") | 185 |
