# Supplementary material for: The problem is escalating: Barriers faced by medical students in conducting research; A scoping literature review
Source: PLoS One. 2026 Feb 27;21(2):e0343232. doi: 10.1371/journal.pone.0343232 (PMC12948103; doi:10.1371/journal.pone.0343232)
Supplement: S3 Appendix — (DOCX) [file pone.0343232.s003.docx]

**S3 Appendix. Data extraction table**

| Author (s), year, & title | Country/ Location | Study design/ method | Population (n) | Barriers identified | Recommendations |
| --- | --- | --- | --- | --- | --- |
| Haran et al., 2023 [1]. *The Current Age of Medical Student Research: A Single-Institution Experience.* | New York | Quantitative study | 577 | - Time limitation - Lack of research knowledge - Difficulties in finding mentors | Reinforcement of research participation. |
| Alhabib et al., 2023 [2]. *Motivators and barriers to research participation among medical students in Saudi Arabia.* | Saudi Arabia | cross-sectional study design | 435 | - Lack of mentoring - Lack of research interest - Lack of time | Raising awareness about the importance of research in academia. |
| Kumar et al., 2019 [3]. *Barriers experienced by medical students in conducting research at undergraduate level.* | Pakistan | Observation, cross-sectional design | 687 | - Lack of research knowledge - Lack of time - Lack of mentoring - Lack of database access - Lack of time - Lack of finances | Improvement of clinical research among undergraduate curriculum. |
| Orebi et al., 2023 [4]. *Medical students’ perceptions, experiences, and barriers towards research implementation at the faculty of medicine, Tanta university.* | Egypt | Cross-sectional design | 462 | - Lack of funding, time, and training in research methods | Improvements in research training and opportunities among undergraduate medical students. |
| Ibrahim Abushouk et al., 2016 [5]. *Attitudes and perceived barriers among medical students towards clinical research: a cross‐sectional study in an Egyptian Medical School.* | Egypt | Cross-sectional design | 420 | - Lack of time, proper mentoring, and funding. | Addressing the research barriers among undergraduate medical students. |
| Alsaleem et al., 2021 [6]. *Challenges and barriers toward medical research among medical and dental students at King Khalid University, Abha, Kingdom of Saudi Arabia.* | Saudi Arabia | Cross-sectional design | 327 | - Lack of time, skills, funding, facilities, and limited access to medical journals and related databases. | Implementation of research training and adequate research support. |
| Quintero et al., 2025 [7]. *Attitudes toward and perceptions of barriers to research among medical students in the context of an educational and motivational strategy.* | Ecuador | Cross-sectional study | 90 | - time constraints, lack of mentorship, and insufficient training in scientific writing and statistics. | Fostering a meaningful research engagement among medical students. |
| Sanabria-de la Torre et al., 2023 [8]. *Medical students’ interest in research: changing trends during university training.* | Spain | Cross-sectional design | 91 | - Insufficient skills to engage in scientific research - Lack of encouragement about the scientific research activities from the professors - lack of funding and lack of awareness of opportunities. | Implementation of research among undergraduate medical students. |
| Dadipoor et al., 2019 [9]. *Barriers to research activities as perceived by medical university students: A cross-sectional study.* | Iran | Cross-sectional | 400 | - Inadequate knowledge of research methodology, inadequate skill in research conduction, and limited access to information sources | Motivating medical students about the importance of research during their training. |
| Hamdan & Kakarla (2013) [12]. *Barriers faced by medical students interested in research.* | Taiwan | Cross-sectional design | 531 | - Lack of opportunities to carry out r4esearch, research not taught - lack of awareness of potential research projects and the lack of time allocated to conduct research | The implementation o0f research modules in medical schools. |
| Ahmad et al., 2022 [13]. *An assessment of publishing practices and barriers faced by medical students to conduct research: A cross‐sectional study from Pakistan.* | Pakistan | Descriptive, cross-sectional design | 1252 | - Lack of mentorship, and lack of research teaching. | The development of policies to facilitate and encourage medical students who are passionate about research. |
| Al Absi et al., 2024 [14]. *Barriers preventing medical trainees from active participation in research activities.* | United Arab Emirates | descriptive cross-sectional design | 57 | - Insufficient statistical and methodology training, the weight of other educational commitments, as well as inadequate incentives and rewards. | Implementation of research support. |
| Hasan et al., 2025 [15]. *Research involvement among undergraduate medical students in Bangladesh: a multicenter cross-sectional study.* | Bangladesh | Cross-sectional design | 2864 | - Insufficient mentorship, and lack of funding. | Promotion oif research engagement among medical students. |
| Pop & Lotrean (2024) [16]. *Comparative analysis of factors and barriers intervening in research participation among romanian and international medical graduates from one romanian medical faculty across three generations.* | Romania | Cross-sectional design | 572 | - Lack of funds and time | Encouraging medical students to participate in research early as possible during their period of training. |
| Mahmood et al., 2025 [17]. *Barriers to undergraduate medical students’ research engagement in Pakistan: a qualitative exploration.* | Pakistan | Qualitative exploratory design | 45 | - Time constraints, as academic workloads and clinical rotations left limited time for research - Lack of extrinsic motivation, knowledge, and research ability, including insufficient training and limited institutional incentives - Inadequate financial assistance and poor maintenance of research facilities, with students highlighting a lack of funding and outdated infrastructure; - Lack of support from research mentors and training | The integration of research in medical curriculum to reinforce the importance of research. |
| Unnikrishnan et al., 2014 [18]. *Medical students’ research–facilitators and barriers.* | India | Cross-sectional design | 105 | - Lack of time, lack of motivation to conduct research | Addressing issues like workload reduction so that medical students have time to learn research. |
| Anbari et al., 2015 [19]. Barriers and challenges in researches by Iranian students of medical universities. | Iran | Mixed method | 627 | - Lack of time, scientific writing skills, and access to trained assistants. | The employment of research experts in the universities to teach research. |
| Narasimhaiah etr al., 2020 [20]. *Attitudes and barriers of medical students towards conducting research in a medical college.* | India | Cross-sectional design | 360 | - Lack of research experience | Provision of sufficient research training and time in medical schools. |
| Chellaiyan et al., 2019 [21]. *Medical research: Perception and barriers to its practice among medical school students of Chennai.* | India | Cross-sectional design | 344 | - Difficulty in choosing topic, difficulty in collecting data, and allocation of time | Addressing the research barriers and support systems that could encourage medical students to engage in research. |
| Alyousefi et al., 2023 [22]. *How do medical students perceive their research experiences and associated challenges?* | Saudi Arabia | Descriptive cross-sectional design | 389 | - Inadequate research supervisors’ guidance and support, lack of time and mentors. | Interventions to promote research mentors to mentor medical students. |
| Assar et al., 2022 [23]. | Saudi Arabia, United Arab Emirates, Egypt, Qatar, Kuwait, & Oman | Cross-sectional design | 2989 | - Lack of time, and lack of access to research lab | Improve research knowledge among medical students. |
| El Achi et al., 2024 [24]. Perception, attitude, practice and barriers towards medical research among undergraduate students. | Beirut | cross-sectional design | 523 | - Lack of time and a lack of knowledge and appropriate skills | The integration of research workshops and training programs into the undergraduate curriculum. |
| Noorelahi et al., 2015 [25]. *Perceptions, barriers, and practices of medical research among students at Taibah College of Medicine, Madinah, Saudi Arabia.* | Saudi Arabia | Cross-sectional design | 233 | - Inadequate facility for research, lack of interest by faculty or guide, and unavailability of the samples or patients. | Addressing perceived barriers and involve medical students in research programmes. |
| *Abusamak et al., 2024 [26]. Knowledge, attitudes, practices and barriers of medical research among undergraduate medical students in Jordan: a cross-sectional survey.* | Jordan | Cross-sectional design | 333 | - Insufficient training in medical research, lack of sufficient research opportunities, and lack of stimulation and support from faculty. | Research modules should be implemented. |
| Sharma et al., 2021 [27]. *Knowledge, attitude, practice and barriers for research amongst medical students of GMC, Nagpur.* | India | Cross-sectional design | 156 | - Lack of time, lack of proper guidance, and lack of funding. | Implementation of scientific research writing. |
| Bashir et al., 2023 [28]. *Obstacles Encountered by Medical Students in Conducting Academic Research-A Cross-Sectional Study.* | Pakistan | Cross-sectional design | 164 | - Limited time, lack of funding, lack of research knowledge, lack of support, and limited sources. | Involvement of medical students in research to better their knowledge. |
| Soe et al., 2018 [29]. *Knowledge, attitudes, and barriers toward research: The perspectives of undergraduate medical and dental students.* | Malaysia | Cross-sectional design | 295 | • Lack of time, lack of knowledge and skills, lack of funding, and lack or rewards. | Creation of support environment to promote research among medical students. |
| Ferdoush et al., 2021 [30]. *Attitude and perceived barriers towards research among undergraduate medical students of Bangladesh.* | Bangladesh | Cross-sectional design | 1279 | • lack of time and priorities to do research work, insufficient guidance, lack of familiarities with research methodology and statistical analysis. | Increase research participation in medical schools. |
| Pallamparthy & Basavareddy (2019) [31]. *Knowledge, attitude, practice, and barriers toward research among medical students: A cross-sectional questionnaire-based survey.* | India | Cross-sectional design | 300 | - lack of research awareness, interest, funds, and time. | Involving medical students in research. |
| Hart et al., 2022 [32]. *Research supervisors’ views of barriers and enablers for research projects undertaken by medical students; a mixed methods evaluation of a post-graduate medical degree research project program.* | Sydney | Mixed method study | 130 | - Lack of protected time for research activities, data acquisition problems | Further support for research and statistics analysis. |
| *Amin et al., 2012 [33]. Knowledge, attitudes and barriers related to participation of medical students in research in three Arab Universities.* | Saudi Arabia, Bahrain, Kuwait | Cross-sectional design | 423 | - shortage of time and a lack of - adequate mentoring. | Reinforce the integration of research into the undergraduate medical curriculum |
| Mokresh et al., 2024 [34]. *Knowledge, attitudes, and barriers toward Research among Medical students: a cross-sectional study in Turkey.* | Turkey | Cross-sectional design | 487 | - Poor level of knowledge regarding research, lack of funding, time, proper mentoring, laboratories, and facilities. | There must be some developments of training systems to convert medical students’ attitudes toward research. |
| Alsulami et al., 2023 [35*]. “Publish or Perish”: barriers to research publication in an undergraduate medical research program.* | Saudi Arabia | Cross-sectional design | 162 | - unsupportive research supervisor, lack of time, inability to reconcile between research and studying | Encouraging medical students to participate in research. |
| AlGhamdi et al., 2014 [36]. *Perceptions, attitudes and practices toward research among senior medical students.* | Saudi Arabia | Cross-sectional design | 172 | - Lack of professional supervisors, lack of time, and lack of funding | Addressing these barriers to better more research interest among the medical students. |
| Turk et al., 2018 [37]. *Attitudes, barriers, and practices toward research and publication among medical students at the University of Damascus, Syri.* | Syri | Cross-sectional design | 323 | - Poor research education, and limited participation | Involve medical students in research projects. |
| Bassey et al., 2023 [38]. *Barriers and challenges to effective medical research among nigerian medical students: a cross-sectional study.* | Nigeria | Cross-sectional design | 421 | - Environmental, practical, academic, and institutional. | Promoting research in academia. |
| Rehman (2024)., [39]. *Barriers to perform research studies from the perspective of medical students of Khyber Medical College, Peshawar*. | Pakistan | Cross-sectional design | 264 | - failure to allocate sufficient funds, | Promote and enhance research culture. |
| Kingpriest et al., 2025 [40]. *A national cross-sectional study on research opportunities and barriers among medical students in Nigeria, with recommendations.* | Nigeria | Cross-sectional design | 429 | - Time constraints, lack of research funding, and inadequate research training | Enhancing structured research opportunities in medical schools. |
|  |  |  |  |  |  |
| Griffin & Hindocha (2011) [41]. *Publication practices of medical students at British medical schools: experience, attitudes and barriers to publish.* | Britain | Cross-sectional design | 515 | - Poor encouragement by seniors to participate in research. | Medical students require to be involved in research projects. |
| Fida et al., 2022 [42]. *The inclination of undergraduate students at King Edward Medical University towards research and its perceived barriers and facilitators; a cross-sectional study.* | Pakistan | Cross-sectional design | 305 | - Academic overload, lack of structured research training, and difficulty in publishing | Implementation of research seminars. |
| Osman (2016) [43]. *Medical students’ perceptions towards research at a Sudanese University.* | Sudan | Cross-sectional design | 104 | - Lack of funding, insufficient time, and the demands of the curriculum | The need to address the research gaps in the medical curriculum. |
| Chenfouh et al., 2024 [44]. *Knowledge, Attitude, and Perceived Barriers of Undergraduate Medical Students Towards Research. A Cross-Sectional Questionnaire-Based Study in Morocco.* | Morocco | Cross-sectional design | 754 | - Time constraints, insufficient funding, inadequate laboratory facilities, and limited understanding of research basics | Enhancing students’ engagement in undergraduate research. |
| Althubaiti et al., 2017 [45]. *Assessment of medical students’ attitudes towards research and perceived barriers.* | Saudi Arabia | Cross-sectional design | 237 | - Lack of time, academic pressure | Introducing the research program in the medical curriculum. |
| Sayed et al., 2022 [46]. *Knowledge, attitude, practices and perceived barriers towards research among the undergraduate medical students of Government Medical College in Rajasthan.* | India | Cross-sectional design | 410 | - Difficulty in understanding the basic research concepts, lack of time, funding, interdepartmental cooperation, motivation, and interest. | Implementation of research course. |
| Abu-Helalah et al., 2015 [47]. *Research participation among medical students in Jordan: rates, attitudes, and barriers.* | Jordan | Cross-sectional design | 1703 | - Insufficient training in medical research, lack of sufficient research opportunities, and lack of stimulation and support from faculty members | Improve research participation among medical students. |
| Ahmed et al., 2023 [48]. *Confronting challenges: an inductive thematic analysis of barriers and solutions to undergraduate medical research in Pakistan.* | Pakistan | Exploratory qualitative design | 33 | - Ineffective research methodology training, lack of access to medical journals and research software | Frequent research workshops and conferences, strong networking, reorienting curriculum to provide early  exposure to research and student-led initiatives were suggested to improve undergraduate research. |
| de Oliveira et al., 2011 [49]. *Student views of research training programmes in medical schools.* | Brazil | Cross-sectional design | 1004 | - Lack of institutional incentive, effective infrastructure and insufficient time available for professors to mentor undergraduate students | Engaging medical students in research. |
| Sabbah et al., 2024 [50]. *Beyond the curriculum: unveiling medical students’ drivers and barriers to research participation at Alfaisal University.* | Saudi Arabia | Mixed method study | 116 | - Lack of hands-on experiences, and mentorship | Emphasize the importance of research. |
| Alexander et al., 2019 [51]. *Perceived barriers to research amongst medical students: Where are we six years on?* | England | Cross-sectional design | 57 | - difficulty in seeking out supervisors, lack of time, and lack of training in research. | Increase awareness of research projects. |
| Yasmin et al., 2022 [52]. *Practice on biomedical research and barriers experienced by the post-graduate medical students in Bangladesh.* | Bangladesh | Cross-sectional design | 325 | - ack of research training and necessary skills, lack of fund, lack of interest and motivation, and lack of appropriate knowledge | Promoting research mentorship. |
| Riva et al., 2019 [53]. *Medical students’ challenges and suggestions regarding research training: a synthesis of comments from a cross-sectional survey.* | Canada | Cross-sectional design | 498 | - Lack of mentorship, and lack of time | Stress out the importance of research in medicine. |
| Baral et al., 2023 [54]. *Knowledge, Attitude, Practice and Barriers Toward Research among Undergraduate Medical Students.* | India | Cross-sectional design | 218 | - Lack of opportunity to conduct research, lack of funding for research | Stress out research participation. |
| Jobran et al., 2024 [55]. *Publication practices of medical students at medical schools in Palestine: experiences, attitudes, and barriers to publishing.* | Palestine | Cross-sectional design | 425 | - Lack of mentorship | Creating long-term investments in research training. |
| Al-saeed et al., 2020 [56]. Barriers toward clinical research perceived by medical students: a descriptive study from Saudi Arabia. | Saudi Arabia | Cross-sectional design | 522 | - Lack of access to counsel and appropriate guide in research, lack of time, lack of funds and mentors, lack of experience with research methods, and insufficient motivation and rewards. | Addressing these barriers and promoting research in medical schools. |
| Jacobs et al., 2022 [57]. *Medical students’ confidence in their abilities and barriers to conducting research: a mixed-methods study.* | USA | Mixed method study | 141 | - Lack of research interest and motivation | Incorporating research with the curriculum. |
