# Supplementary material for: The problem is escalating: Barriers faced by medical students in conducting research; A scoping literature review
Source: PLoS One. 2026 Feb 27;21(2):e0343232. doi: 10.1371/journal.pone.0343232 (PMC12948103; doi:10.1371/journal.pone.0343232)
Supplement: Attachment — Submitted filename: EDITOR RESPONSE-plos One.docx [file pone.0343232.s005.docx]

EDITOR RESPONSE

We sincerely thank you and the reviewers for the constructive feedback provided on our manuscript, “The Problem Is Escalating: Barriers Faced by Medical Students in Conducting Research; A Scoping Literature Review.” We greatly appreciate the time and effort invested in evaluating our work and offering insightful suggestions to enhance its clarity, rigor, and impact.

1. When submitting your revision, we need you to address these additional requirements.

Response: Thank you. We adhered to the Plos One submission guideline.

2. Please ensure that you include a title page within your main document. We do appreciate that you have a title page document uploaded as a separate file. Could you therefore please include the title page at the beginning of your manuscript file itself, listing all authors and affiliations.

Response: The title page is included in the main document.

3. When completing the data availability statement of the submission form, you indicated that you would make your data available on acceptance. We strongly recommend all authors decide on a data sharing plan before acceptance, as the process can be lengthy and hold up publication timelines. Please note that, though access restrictions are acceptable now, your entire data will need to be made freely accessible if your manuscript is accepted for publication. This policy applies to all data except where public depositions would breach compliance with the protocol approved by your research ethics board.

Response: Thank you. This section was revised. Regarding the data availability statement, we would like to clarify that this is a scoping literature review and does not involve the collection of primary or participant-level data. Therefore, this requirement is not applicable to our manuscript. All data synthesized and analyzed are derived from previously published studies, which are properly cited in the manuscript.

4. We note that your Data Availability Statement is currently as follows: All relevant data are within the manuscript and its Supporting Information files.

Response: This part was amended by the authors. It is not applicable in this study.

5. Please amend either the title on the online submission form (via Edit Submission) or the title in the manuscript so that they are identical.

Response: Thank you. We have made amendments.

6. One of the noted authors is a group or consortium. In addition to naming the author group, please list the individual authors and affiliations within this group in the acknowledgments section of your manuscript. Please also indicate clearly a lead author for this group along with a contact email address.

Response: Thank you. We have done that.

7. Your ethics statement should only appear in the Methods section of your manuscript. If your ethics statement is written in any section besides the Methods, please move it to the Methods section and delete it from any other section. Please ensure that your ethics statement is included in your manuscript, as the ethics statement entered into the online submission form will not be published alongside your manuscript.

Response: Thank you. The ethics statement is now moved to the methodology section.

8. We note that you have referenced (Rehman MZ, Yahya AW, Imran J, Shehryar M.) which has currently not yet been accepted for publication. Please remove this from your References and amend this to state in the body of your manuscript: (Rehman MZ, Yahya AW, Imran J, Shehryar M. [Submitted]”) as detailed online in our guide for authors

Response: Thank you. Rehman MZ, Yahya AW, Imran J, Shehryar M. Barriers to perform research studies from the perspective of medical students of Khyber Medical College, Peshawar. Med Biomedical J., 1 (3), 8-15. 2024;3(04). The same study is published.

We note that Figure 1 in your submission contain map images which may be copyrighted. All PLOS content is published under the Creative Commons Attribution License (CC BY 4.0), which means that the manuscript, images, and Supporting Information files will be freely available online, and any third party is permitted to access, download, copy, distribute, and use these materials in any way, even commercially, with proper attribution.

Response: We decided to remove this figure from the manuscript.

10 . If the reviewer comments include a recommendation to cite specific previously published works, please review and evaluate these publications to determine whether they are relevant and should be cited. There is no requirement to cite these works unless the editor has indicated otherwise.

Response: Thank you. Reviewer 1 suggested adding a reference that is already included in the manuscript. We did not add it again to avoid duplication, as it is already cited appropriately and contributes to the relevant discussion.

Furthermore, undermined the study done by: Kingpriest PT, Okpanachi JA, Afolabi SA, Ayorinde MM, Muoghallu OI, Alapa GE, Moses MT, Muhideen B, Musa KM, Zarmai J, Aigbodioh F. A national cross-sectional study on research opportunities and barriers among medical students in Nigeria, with recommendations. BMC Medical Education. 2025 May 16;25(1):713. As the authors of this paper, we are humbly requesting to keep this reference as it continues to strengthen this study due to its relevance and quality.

REVIEWER 1:

We sincerely thank you for your careful review of our manuscript and for your constructive and insightful comments. We greatly appreciate the time and effort you devoted to evaluating our work.

Your suggestions have been extremely helpful in improving the clarity, rigor, and overall quality of the manuscript. We have carefully considered each of your comments and revised the manuscript accordingly. All changes have been addressed point by point in the response document. Thank you again for your valuable feedback and for helping us strengthen our study.

| COMMENTS | REVISION |
| --- | --- |
| The manuscript contains multiple internal numerical contradictions that undermine the credibility of the review process. The PRISMA flow diagram reports 400 records assessed for eligibility and 286 excluded, which mathematically should result in 114 eligible studies; however, only 57 studies are reported as included, with no explanation for the remaining 57 records. This represents a fundamental breakdown in PRISMA transparency. | - Thank you very much. We have revised our Prisma diagram and recalculated all the numerical contradictions. |
| There are inconsistent study counts across analytic sections. While the manuscript repeatedly claims inclusion of 57 studies, the geographical distribution presented in the Results section accounts for only 49 studies, leaving eight studies unaccounted for. Similarly, the study design breakdown lists 49 cross-sectional, 4 mixed-methods, and 2 qualitative studies, totaling 55 rather than 57, with no clarification regarding the missing studies. | - Thank you very much. We have revised. Now the total accounts of 57 included studies. See the red highlight. |
| The exclusion criteria are contradictory across sections. The Methods section states that studies published prior to 2010 were excluded, whereas the PRISMA diagram reports exclusion of studies published prior to 2000. This discrepancy renders the screening framework factually inconsistent and unreliable. | - Thank you very much. We have revised this section. Studies published prior to 2010 were excluded. The Prisma flow diagram is now revised. |
| The Methods indicate that the literature search concluded in March 2025, yet the manuscript cites multiple studies published in April and May 2025. The inclusion of post-search publications contradicts the stated search protocol and compromises methodological integrity. | - Thank you very much and we appreciate it a lot. We have revised this section. |
| The PRISMA diagram reports an excessively high full-text retrieval failure rate, with 90 of 400 eligibility-stage records (22.5%) reportedly inaccessible. This level of retrieval failure undermines claims of a comprehensive review and raises questions about institutional access and search rigor. | - Thank you very much. We have revised the Prisma flow diagram. |
| The manuscript suffers from methodological inconsistency in review type. It alternately describes itself as a scoping review and a narrative review, despite these being distinct methodological approaches with different standards, further confusing the analytic framework. | - Thank you very much. This is a scoping review. We have corrected this section. |
| There are numerous language, typographical, and template errors throughout the manuscript, including incorrect words, unintelligible phrases, misspellings, and unreplaced placeholder text (e.g., “XXX”), which detract from scholarly professionalism. | - Thank you very much. We have corrected the grammar. |
| The visual materials are of inadequate quality for scientific publication. One figure is a low-resolution map explicitly labeled “Powered by Bing,” and another is a generic schematic that adds no analytical content beyond restating textual headings. | - Thank you very much. We decided to remove the figures to enhance the transparency of the paper. |
| Your scoping review identifies 'time constraints,' 'lack of mentorship,' and 'institutional barriers' as key themes. This recently published qualitative study (2025) investigates these exact barriers within a specific medical education context (Pakistan). https://doi.org/10.1186/s12909-025-07185-9 Including this citation would provide up-to-date empirical support for your 'Results' section and allow you to triangulate your scoping review findings with recent qualitative evidence regarding the 'dual-focus nature' of medical training that you discuss." | - Thank you. This source you are suggesting is already included. Please check reference [17] |
| In your 'Study Recommendations' and 'Discussion' sections, you emphasize the need to 'Integrate Research Training Early' and improve research skills. https://doi.org/10.1155/2024/1271802 This study offers a specific pedagogical intervention (peer feedback on research writing) that addresses the 'inadequate training' barrier you identified. Citing this would strengthen your argument for practical, low-resource interventions that institutions can adopt to support student research writing and skill acquisition." | - Thank you. We have added this powerful suggestion. See under discussion section, the last paragraph highlighted in red. |

REVIEWER 2:

We sincerely thank you for your careful review of our manuscript and for your constructive and insightful comments. We greatly appreciate the time and effort you devoted to evaluating our work.

Your suggestions have been extremely helpful in improving the clarity, rigor, and overall quality of the manuscript. We have carefully considered each of your comments and revised the manuscript accordingly. All changes have been addressed point by point in the response document. Thank you again for your valuable feedback and for helping us strengthen our study.

| COMMENTS | REVISION |
| --- | --- |
| 1. While the introduction effectively establishes the importance of research in medical education, the framing remains largely descriptive. The manuscript would benefit from a clearer articulation of: why a scoping review is particularly warranted at this point in time, and how this review advances understanding beyond existing narrative or systematic reviews on student research engagement. Consider sharpening the rationale to explicitly state the gap this review addresses (e.g., fragmentation of evidence, lack of synthesis across regions, or absence of policy-relevant mapping). | - Thank you very much. We have added this suggestion. See the introduction, last paragraph. |
| 2. The author lists databases and keywords used; however, the description remains at a high-level. Boolean operators, truncation, and database-specific adaptations are not described.  Google Scholar is included, but the approach to screening (e.g., number of results reviewed) is not specified. I personally find Google Scholar highly unreproducible for review studies. I would advise you to consider providing a more detailed search strategy (either within the manuscript or as a supplementary file), sufficient to allow replication. | - Thank you very much. We briefly provided the description about Boolean operators. - We decided to remove Google scholar as it is highly unreproducible for reviews. - We have provided a search string labelled as Appendix A attached at the bottom of this manuscript. |
| 3. The review restricts inclusion to English-language studies published between 2010 and 2025. While this is reasonable, the implications of this choice are not discussed on may shape the scope of findings. | - Thank you. We provided the rationale for this. |
| 4. It is unclear how many reviewers conducted screening and whether screening was performed independently or in duplicate. Given the interpretive nature of categorizing “barriers,” this detail is important. Clarify the screening process (number of reviewers, independence of screening, and how disagreements were resolved) to enhance methodological rigor and transparency. | - Thank you. We have revised this section. See under the study selection. |
| 5. The results section identifies several key barriers; however, the analytical process underpinning their identification is not fully elaborated. It is unclear whether thematic synthesis was inductive, deductive, or mixed. The relative weight or frequency of barriers across studies is not described. I think it would be helpful. | - We have added this under data extraction and analysis section. THANK YOU VERY MUCH. |
| 6. The discussion appropriately connects findings to educational reform and institutional responsibility. However, some conclusions appear broader than what the scoping evidence alone can substantiate. consider moderating claims related to policy reform by clearly distinguishing between findings derived from the review and interpretive implications offered by the authors. | - Thank you. We have refined this part. See below the discussion section. |
| Minor grammatical and formatting issues are present and would benefit from careful copy-editing. | - The grammar is now corrected. |
| The abstract could more clearly indicate that this is a scoping review rather than a systematic or effectiveness review. | - We have revised the abstract as you suggested. Thank you. |
| The conclusion may be strengthened by briefly restating the unique contribution of the review rather than reiterating general challenges already described. | - We have revised the conclusion as you suggested. Thank you. |
